# VvERF105 enhances drought resistance in grape through interaction with VvSnRK1

**DOI:** 10.3389/fpls.2026.1884274

**Published:** 2026-07-09

**Authors:** Xiaoyue Cui, Yusui Lou, Ke Zhang, Huiran Lu, Hongquan Shang, Zhongwei Lü, Wenying Wu

**Affiliations:** Institute of Horticultural Research, Henan Academy of Agricultural Sciences, Zhengzhou, China

**Keywords:** CRISPR/Cas9, functional analysis, transgenic, VvERF105, VvSnRK1

## Abstract

**Background:**

Drought stress severely restricts grape growth and yield. *ERF105* is widely involved in plant developmental processes as well as responses to biotic and abiotic stresses. Nevertheless, existing studies of the *ERF105* gene have primarily focused on cold and disease resistance, leaving its potential function in drought response largely unexplored. Therefore, investigating the role of *ERF105* under drought conditions is crucial for understanding the molecular mechanisms of stress tolerance in grape and for breeding drought-resistant cultivars.

**Methods:**

A *VvERF105* gene was cloned from drought-resistant *Vitis vinifera* cv. ‘Thompson Seedless’, followed by sequence analysis, subcellular localization assay, and expression pattern analysis. A dual-target gene editing vector of *VvERF105* was subsequently constructed and transformed into embryogenic calli of ‘Thompson Seedless’ via *Agrobacterium*-mediated genetic transformation. Gene-edited and wild type (WT) grapes were subjected to drought treatment. The biological function of *VvERF105* under drought stress was determined by observing the plant growth status and stomatal aperture, measuring the proline and malondialdehyde (MDA) contents, antioxidant enzyme activities, and the expression levels of drought-related genes. In addition, proteins interacting with VvERF105 were screened and verified using yeast two-hybrid, bimolecular fluorescence complementation (BiFC), and co-immunoprecipitation (Co-IP) assays. Their interaction was further confirmed using *in vitro* phosphorylation assays.

**Results:**

*VvERF105* is a stress-responsive gene localized in the nucleus. It responds to drought, cold, and high-temperature stresses and may act downstream of the ABA signaling pathway. *VvERF105* mutant grapevine plants exhibited reduced resistance to drought stress. The edited lines exhibited smaller stomatal apertures, lower proline content, higher MDA content, and lower antioxidant enzyme activities compared to WT plants under drought stress. The expression levels of *VvDREB2A*, *VvERD14*, *VvKIN2*, *VvNCED1*, *VvRD22*, and *VvRD29B* were also significantly downregulated. VvSnRK1 was identified as an interacting protein of VvERF105 and interacts with it in a phosphorylation-independent manner.

**Conclusions:**

*VvERF105* is a nucleus-localized stress-responsive transcription factor that positively regulates grapevine drought stress responses. Its disruption significantly reduces drought resistance. Moreover, it interacts with the kinase VvSnRK1 in a phosphorylation-independent manner to mediate drought stress signaling in grapevines. The aforementioned results provide valuable genetic resources for molecular breeding of grapevines with enhanced drought resistance.

## 1 Introduction

Grapevine ranks as the world’s fourth major fruit crop and occupies a pivotal position in global agriculture and economy owing to its distinctive flavor, high nutritional value and diverse applications (Zhou et al., 2022). Water is an indispensable limiting factor for grape growth, yield and quality. While mild water deficit benefits the improvement of grape berry quality (Romero-Trigueros et al., 2017), moderate water scarcity causes yield reduction and quality deterioration (Mirás-Avalos and Intrigliolo, 2017), and severe water shortage may even lead to the death of grapevines (Flor et al., 2025).

Notably, drought is a major abiotic stress that limits grapevine growth, development, and productivity, resulting in annual yield losses that exceed those caused by all pathogens combined (Gupta et al., 2020). About 97% of Earth’s water resources are stored as saline water in oceans, while merely 1% of freshwater is accessible for plant utilization (Chen et al., 2021). Agricultural water demand is expected to increase further because of global climate change. Meanwhile, the availability of freshwater remains severely limited, leaving crops around the world increasingly vulnerable to severe drought stress (Gebrechorkos et al., 2025). Drought exerts substantial detrimental impacts on grapevines and the grape industry. Notably, insufficient soil moisture compromises root development and nutrient absorption, leading to stomatal closure, reduced photosynthetic efficiency, and suppressed plant growth (Gambetta et al., 2020). The low temperatures accompanied by dry air and soil during winter induce shoot desiccation, thereby causing severe yield losses in grape production. In contrast, drought disrupts flowering and pollination, resulting in a decreased fruit set rate (Romero-Trigueros et al., 2017). Therefore, mining and utilizing drought-tolerant genes could effectively enhance the drought resistance of grapevines and mitigate yield and quality losses under water-deficit conditions.

In recent years, significant progress has been made in the molecular mechanisms and applications of drought-resistance gene research in grapevines. Notably, multiple genes associated with the regulation of grape drought resistance have been identified (Cardone et al., 2019; Cui et al., 2020; Konecny et al., 2025). Several drought-resistance genes have been successfully transformed into model plants using genetic engineering technology. These phenomena have helped to confirm their functional roles and supported their use in molecular breeding for developing drought-resistant crops. For instance, overexpression of the ‘Yanshan’ grape thioredoxin *VyTRXy* gene improved the drought tolerance of transgenic tobacco by maintaining photosynthesis and enhancing antioxidant and osmotic adjustment ability (Xiang et al., 2023). Similarly, overexpression of the *VhMYB2* gene in *Arabidopsis*, which is from the abiotic stress-resistant rootstock ‘Beta’, increased the resistance of transgenic *Arabidopsis* to salt and drought stress (Ren et al., 2023). In another study, the expression of the *VvWRKY18* gene was induced by drought stress and abscisic acid (ABA). Overexpression of *VvWRKY18* in *Arabidopsis* resulted in increased stomatal density and a higher rate of water loss, ultimately leading to reduced drought tolerance (Zhang et al., 2022). *VviExo70B* plays a role in drought and salt tolerance in grapes through ABA-dependent and ABA-independent pathways (Wang L et al., 2023).

ERF105 belongs to the AP2/ERF (APETALA2/ethylene-responsive factor) family, a large plant-specific transcription factor family composed of five subfamilies: AP2 (APETALA2), RAV (related to ABI3/VP1), ERF (ethylene-responsive factor), DREB (dehydration-responsive element binding protein), and Soloist (Sakuma et al., 2002). Members of the AP2/ERF family participate in both ABA-dependent and ABA-independent, as well as ethylene-dependent signaling pathways (Medina et al., 1999; Li et al., 2023; Ma N et al., 2024). All genes belonging to this family contain an AP2/ERF DNA-binding domain of approximately 60 amino acids, which regulates the transcription of downstream functional genes by binding to the GCC-box (5’-TAAGAGCCGCC-3’) and DRE cis-elements (5’-TACCGACAT-3’) in their promoters (Ohme-Takag and Shinshi, 1995; Stockinger et al., 1997). Notably, these genes are widely involved in plant developmental processes and responses to biotic and abiotic stresses (Nie and Wang, 2023; Ma ZM et al., 2024; Su et al., 2025). Existing studies on *ERF105* function primarily focus on its roles in regulating plant resistance to cold and diseases. For instance, Fragaria × ananassa *FaERF105* confers drought and cold tolerance in *Arabidopsis thaliana* and F. × ananassa by upregulating the antioxidant capacity associated with ROS scavenging (Li et al., 2026). In another study, overexpression of *GauERF105* from *Gossypium australe* in *Arabidopsis* confirmed that it plays a pivotal role in conferring resistance against *Verticillium dahliae* infection (Wang YQ et al., 2023). However, its function in drought tolerance remains largely unexplored. Ghorbani et al. (2019) reported significant upregulation of *AtERF105* in *Arabidopsis* leaves under drought stress, suggesting that *AtERF105* is a drought-inducible gene. In addition, *Arabidopsis* overexpressing *VviERF105* from *Vitis vinifera* exhibited enhanced drought tolerance, while non-embryogenic calli of grapevine showed improved tolerance to osmotic stress (Wang et al., 2022). However, the specific role of the *ERF105* gene in drought resistance of grapevine has not been reported. The functional elucidation of the *ERF105* gene in grape drought resistance will enrich the drought-resistant gene resources of grapevines, and help improve grape drought tolerance and achieve water-saving cultivation.

In this study, the *VvERF105* gene was isolated from the drought-resistant grape cv. ‘Thompson Seedless’, and its role in grapes was further investigated using a gene editing approach. The interaction proteins of VvERF105 were also screened and verified. The findings of this study reveal the biological function and molecular mechanism of *VvERF105* in regulating drought resistance of grapes. They form a basis for developing novel drought-resistant grape germplasms by providing important genetic resources for breeding drought-resistant grapes.

## 2 Materials and methods

### 2.1 Plant materials, growth conditions, and stress treatments

Wild-type *Arabidopsis thaliana* (Columbia ecotype, Col-0) and *Nicotiana benthamiana* were grown under a controlled environment at the Institute of Horticultural Research, Henan Academy of Agricultural Sciences. The growth conditions were set at 25 °C and a 16 h light/8 h dark cycle. Ten-year-old ‘Thompson Seedless’ grapevines were maintained at Henan Modern Agricultural Research and Development Base (Yuanyang, Henan, China). Mature and young leaves, tendrils, inflorescences, berries, and stems of untreated ‘Thompson Seedless’ grapes were collected for tissue expression analysis. To explore the expression profile of *VvERF105* under abiotic stress and ABA treatment, the functional leaves at the 3^rd^ and 4^th^ nodes of shoots of ‘Thompson Seedless’ with consistent growth were cut off and preserved in ice. The petioles were wrapped with wet cotton and placed on a tray containing moisturizing filter paper. Leaves were sprayed with 10% polyethylene glycol (PEG) 6000, 200 μM ABA and cultured at 4 °C (cold treatment) and 40 °C (heat treatment). Samples were taken at 0, 2, 4, 8, 12, and 24 h post-treatment, with three biological replicates at each time point. The collected samples were immediately frozen in liquid nitrogen and stored at -80 °C.

Six-week-old *VvERF105* mutant and WT grapevines were watered to saturation and then subjected to drought treatment by withholding irrigation for 24 days. Then, all treated plants were re-watered for recovery. The phenotypic changes were documented photographically before and after the induction of drought.

### 2.2 RNA extraction and qRT-PCR analysis

Total RNA was extracted from the leaves of ‘Thompson Seedless’ grapevines using a plant RNA extraction kit (OMEGA, Norcross, USA). RNA quality was determined using UV spectrophotometry. High-quality RNA (1.8 ≤ OD260/OD280 ≤ 2.0) was reverse transcribed into cDNA using a reverse transcription kit (Tiangen, Beijing, China) in accordance with the manufacturer’s instructions. The qRT-PCR reaction volume was 20 μL, comprising 1 μL of cDNA template (100 ng/μL), 0.8 μL of upstream and downstream primers (10 μM), 10 μL of SYBR Green fluorescent dye, and 7.4 μL of ddH_2_O. The qRT-PCR cycle program was set as follows: initial denaturation at 95 °C for 1 min, followed by 40 cycles of denaturation, primer annealing, and extension at 95 °C for 10 s, 58 °C for 20 s, and 72 °C for 20 s, respectively. Each treatment was replicated thrice. The relative gene expression was analyzed using the 2^–ΔΔCT^ method, with the *VvActin7* gene (accession no. XM_002282480) used as the internal reference. Statistical analysis was done using SPSS 23.0 software (Landau and Everitt, 2003). One-way ANOVA and Tukey’s-b test (*P* < 0.05) were used to evaluate the statistical significance.

### 2.3 Isolation and sequence analysis of *VvERF105*

The coding sequence (CDS) of the *VvERF105* gene was cloned using *VvERF105*-F and *VvERF105*-R (**Supplementary Table 1**) primers and the cDNA derived from ‘Thompson seedless’ leaves as the template. Chromosomal location of the *VvERF105* gene was predicted using EnsemblPlants (http://plants.ensembl.org/index.html), followed by analysis of the conserved domain using SMART (http://smart.embl-heidelberg.de/). Phylogenetic analysis was performed using the maximum likelihood method with 1000 bootstraps in MEGA software (Tamura et al., 2013).

### 2.4 Construction of *VvERF105*-GFP vector and subcellular localization analysis

The CDS of the *VvERF105* gene (excluding stop codon) was constructed into the pCambia2300-GFP vector to form *VvERF105*-GFP fusion expression vector using *VvERF105*-GFP-F and *VvERF105*-GFP-R (**Supplementary Table 1**) primers. An empty vector pCambia2300-GFP was used as the control. The fusion vector and empty-vector plasmids were subsequently extracted and transformed into *Arabidopsis* protoplasts using the kit method (Coolaber, Beijing, China). The transformed protoplasts were observed under a Revolution ECHO microscope to determine the subcellular localization of the VvERF105 protein.

### 2.5 CRISPR/Cas9 vector construction for *VvERF105* gene editing

Target analysis and primer design were done using the online software CRISPR-GE (http://skl.scau.edu.cn). Activation of *Agrobacterium* cells carrying the pYLCRISPR/Cas9Pubi-N vector and the intermediate vector containing pYLgRNA was then carried out. Plasmids were subsequently extracted using the plasmid extraction kit (OMEGA, Norcross, USA). The target was introduced between the promoter and the sgRNA backbone by overlapping PCR to form a sgRNA expression cassette using primers with target sequences (*VvERF105*-gRT1, *VvERF105*-AtU3dT1, *VvERF105*-gRT2, and *VvERF105*-AtU3dT2). The PCR products were subsequently examined by agarose gel electrophoresis. Two sgRNA expression cassettes were cloned into the pYLCRISPR/Cas9Pubi-N vector using the Golden Gate cloning method. The construct was then transformed into *DH10B* cells, and positive single colonies were selected and confirmed by PCR analysis. The primers used are outlined in **Supplementary Table 1**.

### 2.6 Generation and confirmation of gene edited grape

Calli from stem segments of ‘Thompson Seedless’ grape were subjected to *Agrobacterium*-mediated transformation (Cui et al., 2022). The callus was infected with GV3101 carrying plasmid *VvERF105*-T1-T2-pYLgRNA-pYLCRISPR/Cas9Pubi-N. Each infected callus was subsequently cultured on Murashige & Skoog (MS) basal medium containing 30 g/L sucrose, 4 mg/L benzylaminopurine (6-BA), 0.02 mg/L α-naphthalene acetic acid (NAA), 7 g/L agar, 300 mg/L ceftiofur, 200 mg/L carbenicillin, and 75 mg/L kanamycin for 2 months. Subculturing was done once a month. The resistant callus was then induced into form seedlings on differentiation medium (MS + 30 g/L sucrose + 2 mg/L 6-BA + 0.2 mg/L NAA + 7 g/L agar + 300 mg/L ceftiofur + 200 mg/L carbenicillin + 75 mg/L kanamycin). The induced seedlings were transferred to Lloyd & McCown wood plant basic medium (WPM) containing 30 g/L sucrose, 0.2 mg/L 6-BA, 0.2 mg/L indole-3-butyric acid (IBA), 7 g/L agar, and 1.5 g/L activated carbon for further culture.

The target sequence amplification and sequencing primers (*VvERF105*-target-F and *VvERF105*-target-R) were subsequently designed using the primerDesign tool of CRISPR-GE after obtaining the positive plants. The genomic DNA of the positive plant leaves was extracted as a template for cloning. The target fragment was subjected to PCR amplification, and the PCR products were sent to the Shanghai Shenggong Biotechnology Co., Ltd. for sequencing.

### 2.7 Stomatal aperture measurement and biochemical indicator assays

Stomata were visualized using the transparent tape method (Cui et al., 2022). Pore widths were measured using a Nano Measurer. The average value from nine stomata pores per plant (three plants in total) was recorded as the stomatal aperture.

Biochemical indices were quantified using previously reported protocols, with all detection kits obtained from Jiangsu AIDISHENG (Xu et al., 2023; Yu et al., 2026). Proline content was assayed with a proline detection kit (Cat. No. ADS-F-AJS004-96), and malondialdehyde (MDA) content was determined using an MDA kit (ADS-F-YH002-96), both following the manufacturer’s manuals. Activities of superoxide dismutase (SOD), peroxidase (POD), and catalase (CAT) were separately measured with their corresponding commercial kits (SOD: ADS-F-KY011-96; POD: ADS-F-KY003-96; CAT: ADS-F-KY002-96) as instructed by the product specifications.

### 2.8 Self-transcriptional activation and yeast two-hybrid assays

The CDS of *VvERF105* was first cloned into the pGBKT7 vector to serve as a bait vector to screen for proteins interacting with VvERF105. The recombinant plasmid pGBKT7-*VvERF105* was then transformed into the yeast strain Y2H-Gold via the PEG/LiAc method and used to perform the bait vector autoactivation assay. A yeast two-hybrid screen was constructed by co-transforming the bait vector with the yeast cDNA library plasmid into Y2H-Gold competent cells after confirming that the bait vector exhibited no autoactivation activity. The co-transformed cells were plated on SD/-Leu/-Trp/-His/-Ade/AbA selective medium, where only yeast cells harboring both the bait plasmid and library plasmids encoding interacting proteins could survive and form colonies. Yeast single colonies with a diameter greater than 2 mm were picked for PCR-based detection of the colony after 3 days of incubation at 30 °C. Clones with PCR products larger than 500 bp were selected for Sanger sequencing. The obtained sequences were subjected to a homology search through BLAST analysis against the NCBI database (https://www.ncbi.nlm.nih.gov/) to identify candidate genes encoding VvERF105-interacting proteins. The stress-responsive gene *VvSnRK1* was chosen among the candidates for further validation.

### 2.9 Bimolecular fluorescence complementation

The *VvERF105* and *VvSnRK1* genes were separately constructed into the bimolecular fluorescence complementation (BiFC) vectors pEYFP-N and pEYFP-C, respectively, to verify the interaction between VvERF105 and VvSnRK1. The fusion vector plasmids were extracted and purified in large quantities. The recombinant plasmids (pEYFP-N-*VvERF105* + pEYFP-C-*VvSnRK1*, pEYFP-N-*VvERF105* + pEYFP-C, and pEYFP-N + pEYFP-C-*VvSnRK1*) were then co-transformed into *Arabidopsis* protoplasts via the PEG-mediated method. YFP fluorescence was subsequently observed using a laser scanning confocal microscope after incubation under low-light conditions for 20–24 hours.

### 2.10 Co-immunoprecipitation assay

The CDS of *VvERF105* and *VvSnRK1* were fused with an HA tag and a Flag tag, respectively, and cloned into the pCAMBIA1300 vector for Co-IP analysis. The recombinant plasmids were transformed into *Agrobacterium tumefaciens* strain GV3101. *Agrobacterium* suspensions harboring *VvERF105*-HA and *VvSnRK1*-Flag were co-infiltrated into the leaves of 4-week-old *N. benthamiana* plants. Total proteins were subsequently extracted from the infiltrated leaves using ice-cold extraction buffer (50 mM Tris-HCl, pH 7.5, 150 mM NaCl, 1 mM EDTA, 1% Triton X-100, 10% glycerol, 1 mM PMSF, and 1 × protease inhibitor cocktail) at 60 h post-infiltration.

The protein extracts were incubated with anti-HA magnetic beads at 4 °C for 4 hours, with gentle rotation. The beads were then washed thrice using washing buffer (50 mM Tris-HCl, pH 7.5, 150 mM NaCl, 1 mM EDTA, 0.1% Triton X-100) to remove non-specifically bound proteins. The immunoprecipitated proteins were eluted by boiling the beads with 2 × SDS loading buffer. The eluted proteins were subsequently separated on a 10% SDS-PAGE gel and transferred onto PVDF membranes. Western blotting was performed using anti-HA and anti-Flag primary antibodies, followed by HRP-conjugated secondary antibodies. The signals were detected using an enhanced chemiluminescence (ECL) detection system.

### 2.11 Phosphorylation assay *in vitro*

The full-length CDS of *VvSnRK1* was cloned into the pGEX-4T-1 vector to generate the GST-tagged recombinant plasmid (*VvSnRK1*-GST). In contrast, the full-length CDS of *VvERF105* was inserted into the pET-32a vector to construct the HIS-tagged recombinant plasmid (*VvERF105*-HIS). The recombinant plasmids were then transformed into *Escherichia coli* BL21 (DE3) competent cells. The expression of recombinant proteins was induced using 0.5 mM IPTG at 16 °C for 16 h. The proteins were subsequently purified using GST-Sepharose beads (for *VvSnRK1*-GST and GST) and Ni-NTA agarose beads (for *VvERF105*-HIS) according to the manufacturer’s instructions.

For the *in vitro* phosphorylation reaction, purified 1 μg *VvERF105*-HIS and 2 μg *VvSnRK1*-GST proteins were mixed in 1 × kinase buffer containing 50 mM Tris-HCl (pH 7.5), 150 mM NaCl, 2 mM MgCl_2_, 2 mM MnCl_2_, 2 × Phos-tag, 1 mM DTT, and 1 mM PMSF, with ATP supplemented at a final concentration of 1 mM. The reaction mixture was incubated at 30 °C for 1 hour to allow phosphorylation. The reaction was terminated by the addition of 5 × SDS loading buffer. Protein samples were then separated by 10% conventional SDS-PAGE and 10% Phos-tag™ SDS-PAGE, respectively. Finally, phosphorylated protein bands were visualized by Coomassie Brilliant Blue staining.

## 3 Results

### 3.1 Cloning and sequence analysis of *VvERF105*

**Figure 1:**
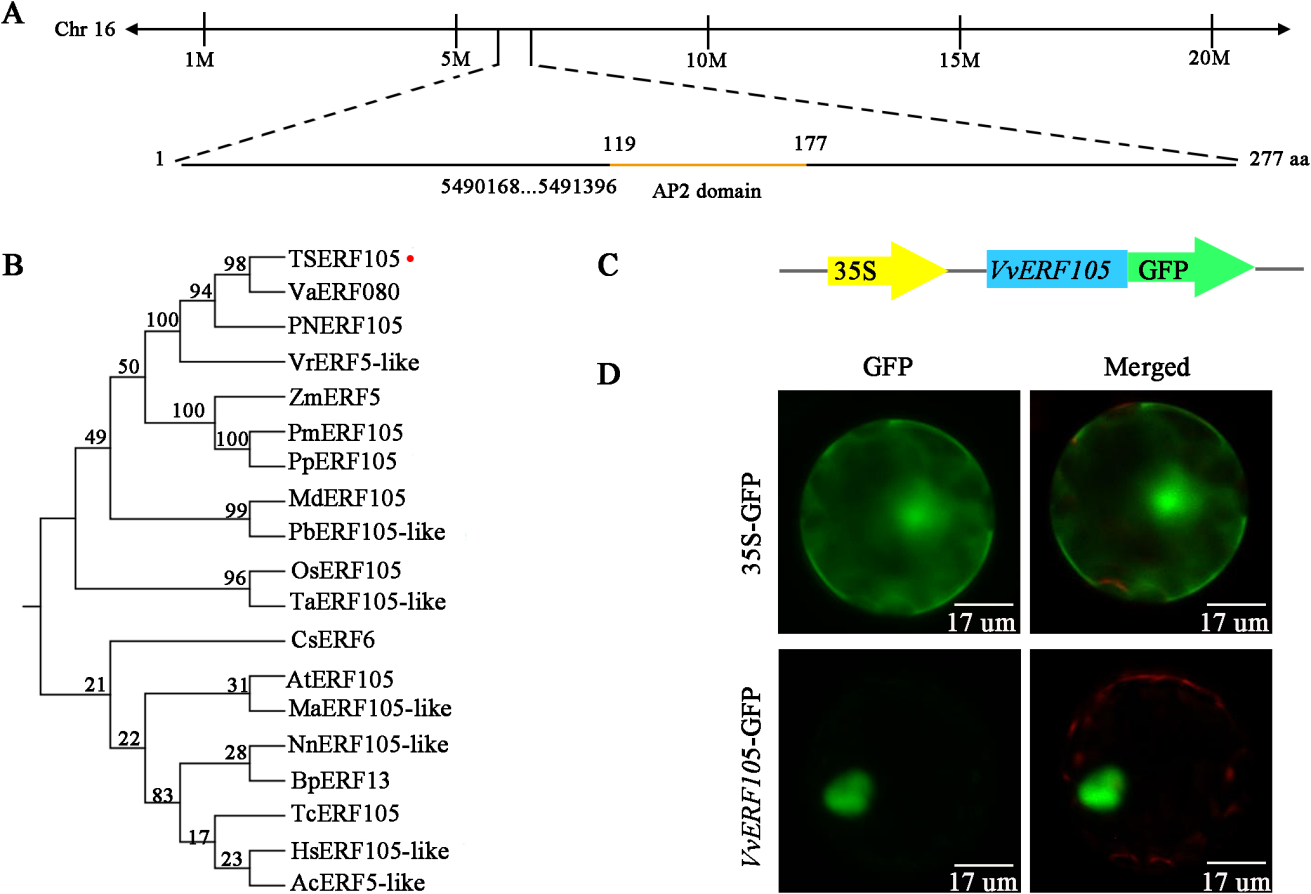
Characterization of VvERF105. **(A)** Chromosomal distribution and conserved domain structure of *VvERF105*. The AP2 domain is marked with orange lines. **(B)** Phylogenetic analysis of VvERF105 and its homologs across plant species, bootstrap values are shown on branches. VvERF105 from ‘Thompson Seedless’ is marked with red dots. TSERF105 (Thompson Seedless), VaERF080 (*V. amurensis*), PNERF105 (Pinot Noir), VrERF5-like (*V. riparia*), ZmERF5 (*Zea mays*), PmERF105 (*Prunus mume*), PpERF105 (*P. persica*), MdERF105 (*Malus domestica*), PcERF105-like (*Pyrus communis*), OsERF105 (*Oryza sativa*), TaERF105-like (*Triticum aestivum*), CsERF6 (*Citrus sinensis*), AtERF105 (*Arabidopsis thaliana*), MaERF105-like (*Musa acuminata*), NnERF105-like (*Nelumbo nucifera*), BpERF13 (*Betula platyphylla*), TcERF105 (*Theobroma cacao*), HsERF105-like (*Hibiscus syriacus*), and AcERF5-like (*Actinidia chinensis*). The accession numbers used are shown in **Supplementary Table 2**. **(C)** Schematic diagram of the VvERF105-GFP fusion expression vector. **(D)** Subcellular localization of VvERF105 protein in *Arabidopsis* protoplasts. Merged panels show combined GFP and chloroplast autofluorescence. Scale bar = 17 μm.

The CDS of *VvERF105* (accession no. OM025222.1) was 834 bp in length, encoding 277 amino acids. It was located on chromosome 16, with a total length of 1,229 bp, and contained a typical AP2 domain (**Figure 1A**). Cluster analysis revealed that VvERF105 was closely related to homologous proteins in *Vitis amurensis*, *V. vinifera* cv. ‘Pinot Noir’, and *V. riparia* (**Figure 1B**). Subcellular localization results revealed that GFP was distributed throughout the cell in the protoplasts of *Arabidopsis thaliana* transformed with pCambia2300-GFP empty vector. In contrast, GFP was only localized in the nucleus in the protoplasts of the transformed recombinant plasmid *VvERF105*-GFP (**Figure 1C**), indicating that VvERF105 plays a role in the nucleus.

### 3.2 *VvERF105* is a stress-responsive gene

**Figure 2:**
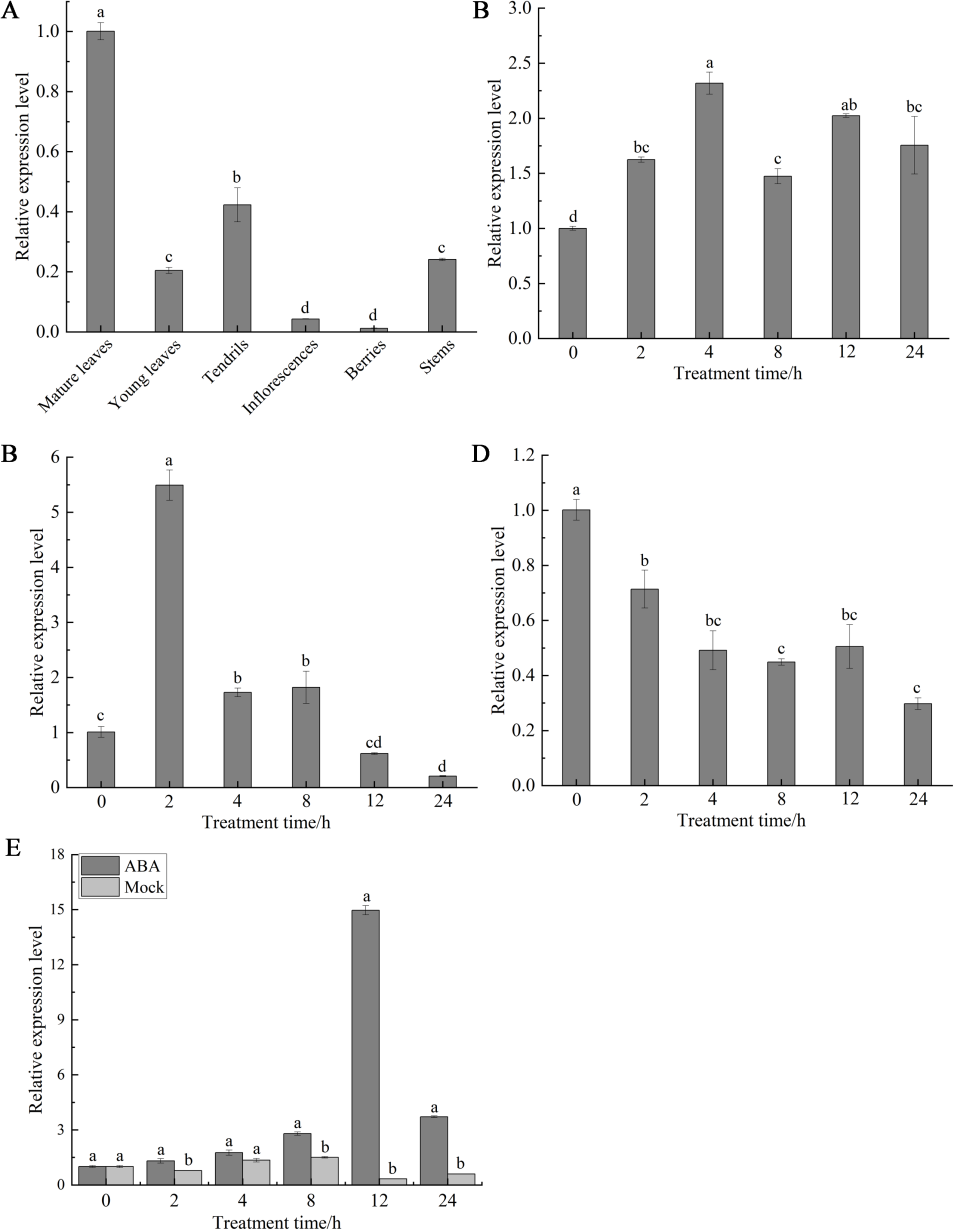
Expression pattern of the *VvERF105* gene in ‘Thompson Seedless’. **(A)** Expression pattern of *VvERF105* in mature leaves, young leaves, tendrils, inflorescences, berries, and stems. **(B)** Expression pattern of *VvERF105* induced by 10% PEG6000 treatment. **(C)** Expression pattern of *VvERF105* induced by cold treatment (4°C). **(D)** Expression pattern of *VvERF105* induced by heat treatment (40°C). **(E)** Expression pattern of *VvERF105* induced by 200 μM ABA treatment. Mock represented H_2_O. Error bars refer to ± SE (n=3). Significance was analyzed by the method of one-way ANOVA followed by Tukey’s-b (K) test. Different lowercase letters denote significant differences at *p* < 0.05. The same below.

The *VvERF105* gene was expressed in different tissues of the ‘Thompson Seedless’ grape. The highest expression was detected in mature leaves, while the lowest expression was detected in berries (**Figure 2A**). The *VvERF105* gene significantly responded to abiotic stress. Notably, the expression level of *VvERF105* under PEG-simulated drought stress reached a peak at 4 h (**Figure 2B**). However, its expression level under low temperature stress first increased and then decreased, reaching the maximum at 2 h after treatment, which was about 5.5-fold that before treatment (**Figure 2C**). The expression of *VvERF105* was inhibited under high temperature stress, with the expression level gradually decreasing with increasing treatment time (**Figure 2D**). The result showed that *VvERF105* is strongly induced by ABA, peaking at 12 h (**Figure 2E**). In summary, the *VvERF105* gene has different expression patterns in different grape tissues. It responds to drought, low temperature, high temperature stresses and exogenous ABA treatment.

### 3.3 Acquisition of *VvERF105* gene editing lines

Two targets were designed at 202–221 bp and 322–341 bp in the CDS region of the *VvERF105* gene. The promoter, target, and sgRNA were constructed into a complete expression cassette, and then integrated into the pYLCRISPR/Cas9Pubi-N vector by edge cutting and ligation (**Supplementary Figures 1A–D**). Three *VvERF105* gene-edited grape lines were obtained through *Agrobacterium*-mediated genetic transformation (**Supplementary Figure 1E**). The target was sequenced, followed by comparing the sequencing results with those of the wild type (WT). Notably, the T1 line had a 15-base deletion in target 1, the T2 line had a 1-base insertion in the downstream of target 1, while the T3 line had a 1-base substitution in the downstream of target 1. Different numbers of base substitutions occurred in the three lines at the position of target 2 (**Supplementary Figure 1F**).

### 3.4 Knockout of the *VvERF105* gene reduced the resistance of grapes to drought stress

**Figure 3:**
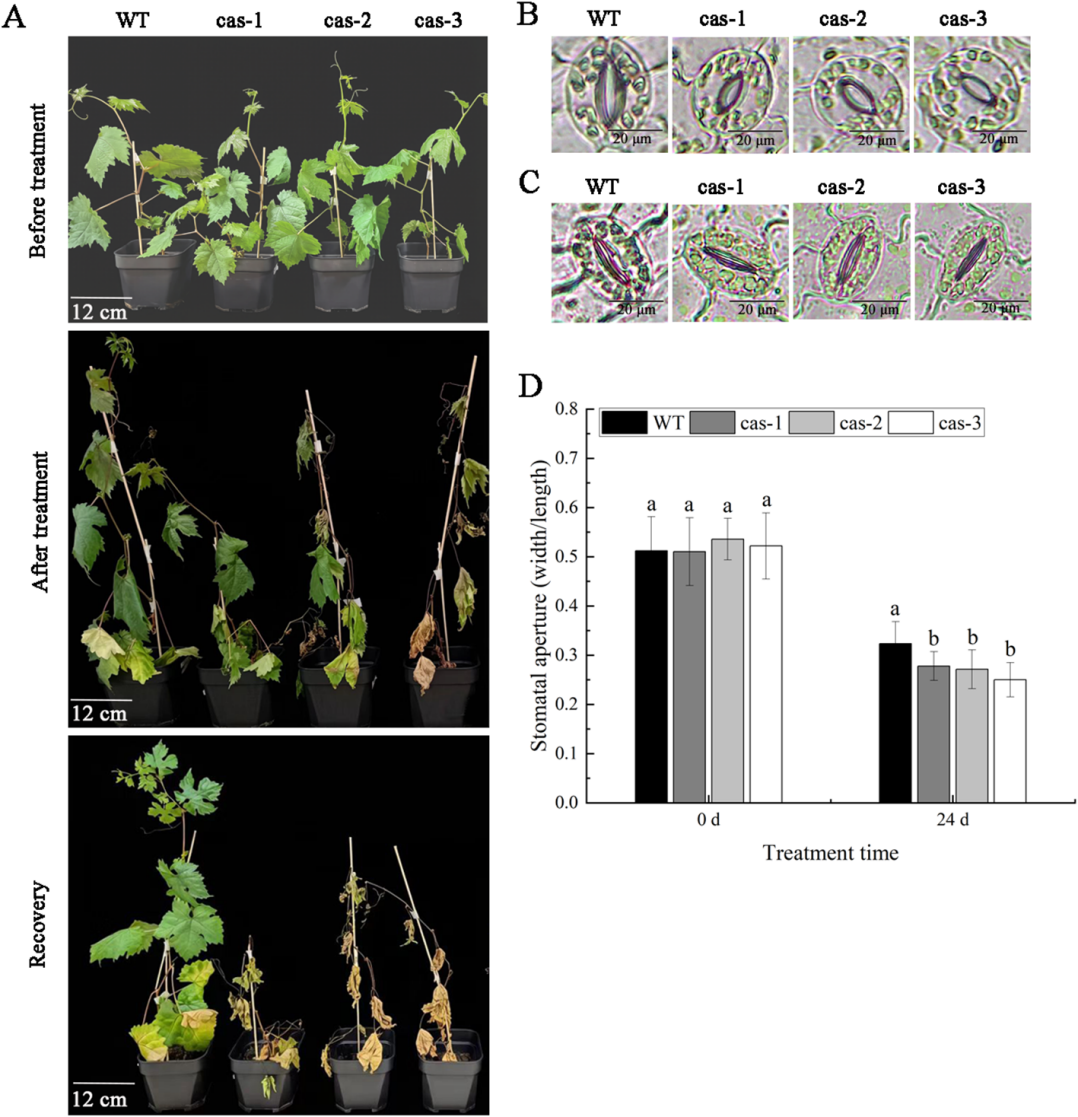
Drought phenotypes and stomatal traits of WT and *VvERF105* gene-edited grapevines. **(A)** Phenotypes of WT and *VvERF105* gene-edited grapevines before and after drought treatment and recovery. Scale bar = 12 cm. **(B, C)** Micrographs of stomatal morphology in WT and *VvERF105* gene-edited lines at 0 d **(B)** and 24 d of drought treatment **(C)**. Scale bar = 20 μm. **(D)** Statistical analysis of stomatal aperture (width/length ratio) in WT and gene-edited plants at 0 d and 24 d post-drought exposure.

**Figure 4:**
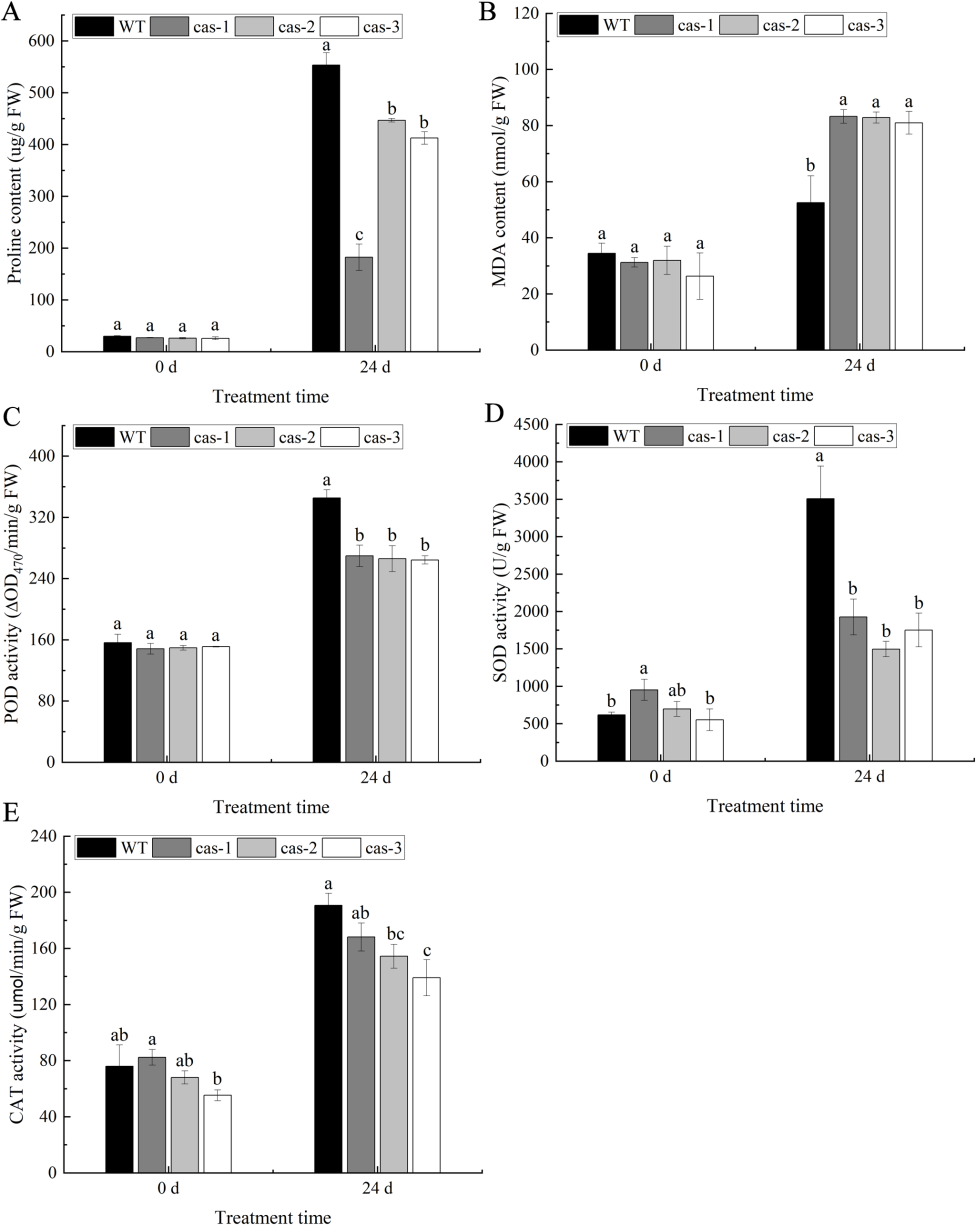
Changes in biochemical indices of WT and *VvERF105* gene-edited grapevines under drought stress. **(A)** Proline content. **(B)** MDA (malondialdehyde) contents. **(C)** POD (peroxidase) activity. **(D)** SOD (superoxide dismutase) activity. **(E)** CAT (catalase) activity.

The three grape lines (edited lines) whose *VvERF105* gene was silenced and the WT lines exhibited significantly different drought resistance phenotypes in the simulated drought stress environment of water control treatment for 24 days. The leaf wilting degree of the three edited lines of the *VvERF105* gene was more severe than that of the WT. Notably, almost all the edited lines died after 7 days of rewatering, while the WT plants returned to normal growth (**Figure 3A**). The tolerance of grapes to drought stress decreased after editing the *VvERF105* gene. No significant differences in the stomatal aperture were observed across all plants before treatment (**Figure 3B**). However, the stomatal aperture of the edited lines was significantly smaller than that of the WT plants after 24 days of drought treatment (**Figures 3C, D**). Under well-watered conditions, proline and MDA contents were comparable among the four lines. Drought stress induced a marked increase in both proline and MDA contents of all plants. Nonetheless, the proline content of the edited lines was lower than that of the WT lines (**Figure 4A**), while the MDA content was higher than that of the WT lines (**Figure 4B**). POD, SOD, and CAT activities increased significantly after drought treatment (**Figures 4C–E**), with the activities significantly lower in the edited lines compared to the corresponding activities in the WT lines.

### 3.5 Knockout of the *VvERF105* gene inhibits the induction of drought-responsive genes

**Figure 5:**
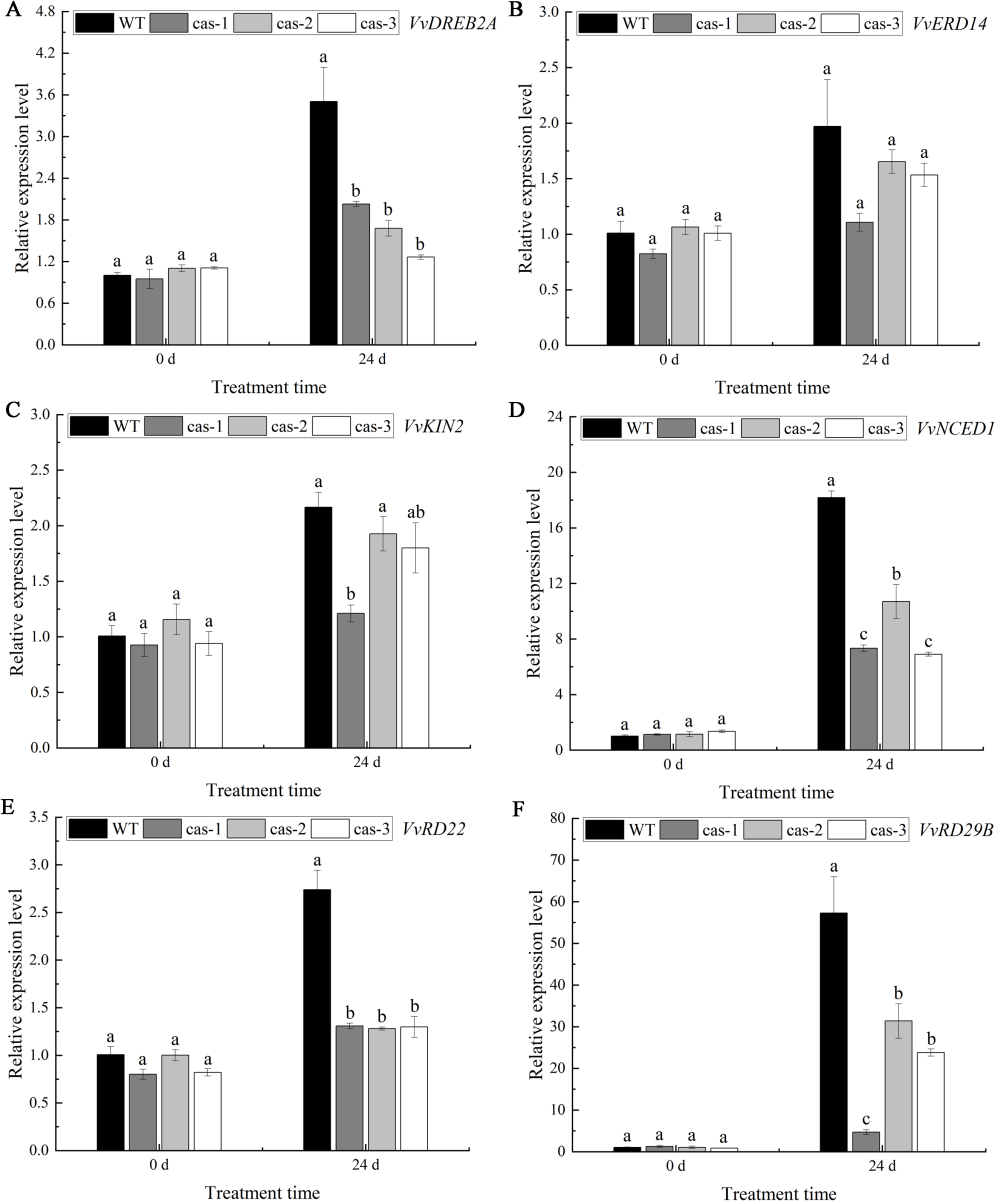
Expression levels of the six drought-related genes of WT and *VvERF105* gene-edited grapevines under drought stress. **(A)** *VvDREB2A*. **(B)** *VvERD14*. **(C)** *VvKIN2*. **(D)** *VvNCED1*. **(E)** *VvRD22*. **(F)** *VvRD29B*.

There was no significant difference in the transcriptional expression levels of six drought-responsive genes, including *VvDREB2A* (accession no. XM_002273802.3), *VvERD14* (XM_002285883.3), *VvKIN2* (XM_002270462.4), *VvNCED1* (NM_001281270.1), *VvRD22* (XM_010650245.3), and *VvRD29B* (XM_059733739.1), between the edited lines and the WT lines under normal growth conditions. This finding indicated that editing of the *VvERF105* gene did not significantly change the basic expression patterns of genes associated with drought resistance under non-stress conditions. In contrast, the six genes associated with drought resistance were significantly upregulated in all lines after 24 days of drought stress treatment. However, the expression level in the *VvERF105* edited lines was significantly lower than that in the WT lines, where the overall expression level was significantly inhibited (**Figure 5**).

### 3.6 VvSnRK1 interacts with VvERF105 but does not phosphorylate it *in vitro*

**Figure 6:**
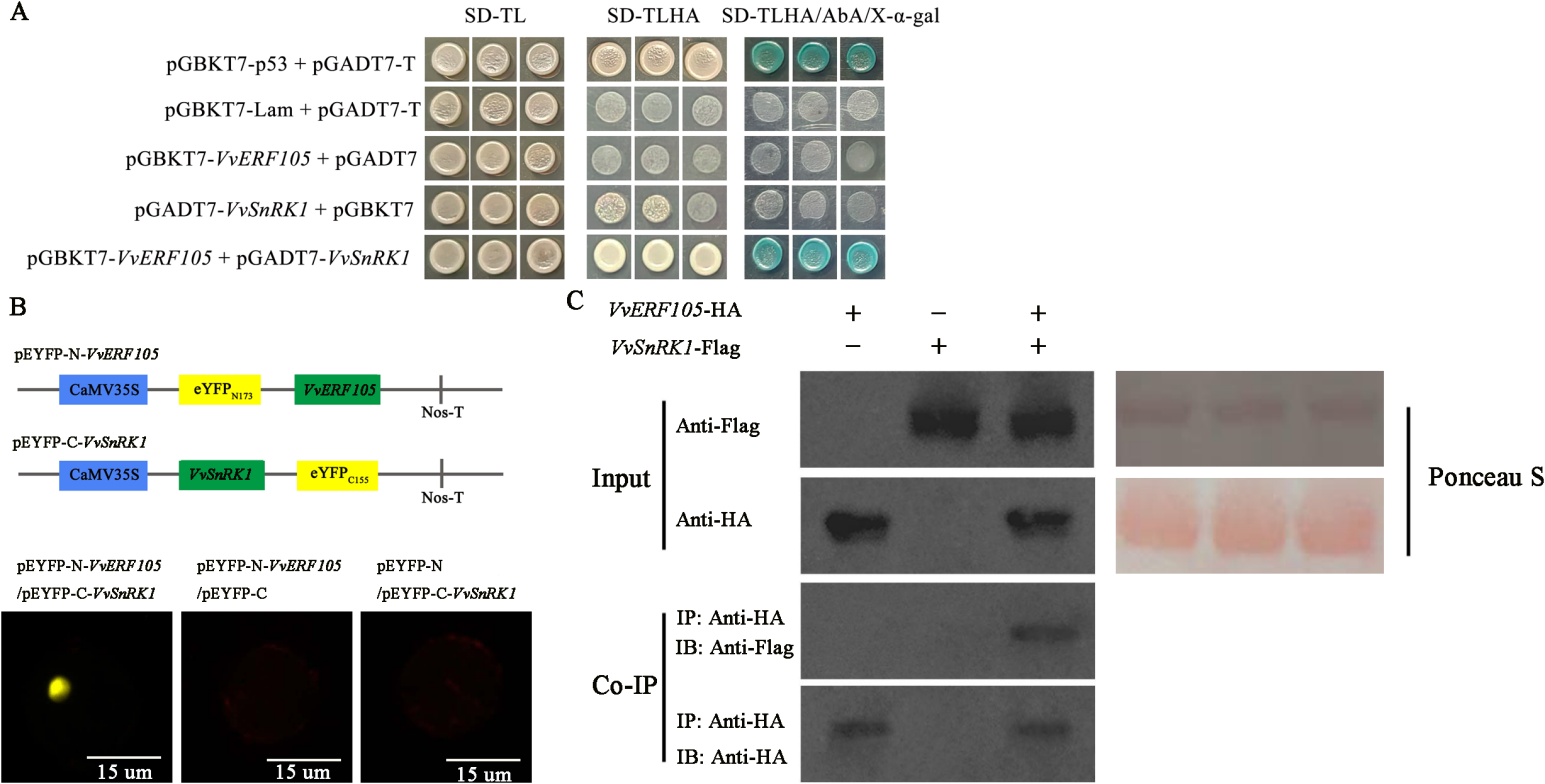
Interaction of VvERF105 and VvSnRK1. **(A)** Interaction of VvERF105 and VvSnRK1 in yeast cells. **(B)** Interaction of VvERF105 and VvSnRK1 in *Arabidopsis* protoplasts. Scale bar = 15 μm. **(C)** Co-immunoprecipitation (Co-IP) assay verifying the physical interaction between VvERF105 and VvSnRK1 in *N. benthamiana*. Proteins were extracted from leaves expressing *VvERF105*-HA and/or *VvSnRK1*-Flag as indicated (+/+: co-expression; +/- or -/+: single controls). Input was analyzed by Ponceau S (loading control) and anti-HA blot. For Co-IP, proteins were immunoprecipitated (IP) with anti-HA, then immunoblotted (IB) with anti-Flag (top) to detect co-precipitated *VvSnRK1*-Flag, and with anti-HA (bottom). *VvSnRK1*-Flag was specifically co-immunoprecipitated only in the co-expression sample (+/+), confirming their interaction.

The self-activation test of the bait vector showed that the recombinant bait plasmid pGBKT7-*VvERF105* could not grow normally on the SD/-Trp/-Leu/AbA/X-α-Gal-deficient screening plate when transferred into the Y2H Gold strain (**Supplementary Figure 2**). There was no blue color reaction, indicating that the VvERF105 protein did not have the transcriptional activity of autonomously activating the yeast reporter gene. On this basis, the bait plasmid pGBKT7-*VvERF105* and the library plasmid were co-transformed into the yeast Y2H strain. Monoclonal yeast spots with a diameter greater than 2 mm were selected after plate screening for PCR detection and sequencing. BLAST analysis of the sequence obtained in NCBI yielded a candidate gene, SNF1-related protein kinase regulatory subunit beta-1 (*VvSnRK1*, accession no. XM_002270647.5). The interaction between VvERF105 and VvSnRK1 was proved by yeast two-hybrid (**Figure 6A**), BiFC (**Figure 6B**), and Co-IP (**Figure 6C**) assays.

*In vitro* kinase assays were carried out to explore whether the interaction between VvSnRK1 kinase and VvERF105 transcription factor was achieved by modifying phosphorylation. Phos-tag SDS-PAGE results revealed no significant change in the migration rate of VvERF105 protein irrespective of whether VvSnRK1 kinase and ATP were added. Notably, no delayed band of phosphorylation modification was detected (**Supplementary Figure 3A**). Conventional SDS-PAGE verified that the amount of protein loading in each group was consistent (**Supplementary Figure 3B**). These results indicate that VvSnRK1 kinase interacts with VvERF105 transcription factors. However, VvSnRK1 does not directly phosphorylate VvERF105, suggesting that the regulatory relationship between the two does not depend on the classical kinase-substrate phosphorylation pathway.

## 4 Discussion

Grapes are a sedentary organism affected by various environmental stress factors. As such, they have developed complex defense strategies to cope with many biotic and abiotic stress factors (Cardone et al., 2019). Transcription factors play a vital role in drought stress response by binding to gene promoters and regulating the expression of specific genes. Several transcription factor families, including MYB (Ren et al., 2023), bZIP (Tu et al., 2016), WRKY (Zhu et al., 2017), NAC (Jin et al., 2025), AP2/ERF (Wang et al., 2022), and bHLH (Wang et al., 2016), harbor drought resistance functions in grapes. AP2/ERFs are a large class of transcription factors mainly present in plants. These transcription factors play important regulatory roles in many biological and physiological processes, such as plant morphogenesis, response mechanisms to various stresses, hormone signal transduction, and metabolite regulation (Feng et al., 2020; Ma ZM et al., 2024). Notably, 149 AP2/ERF genes, including 120 in ERF subfamilies, have been identified from grapes (Licausi et al., 2010). VvERF63 is an ethylene response factor that plays a role in cold resistance by increasing the activity of antioxidant enzymes and the expression level of stress response genes in grape leaves and *Arabidopsis* (Li et al., 2023). The *VaERF092* gene derived from the amur grape confers cold tolerance in *Arabidopsis* by regulating the expression level of *VaWRKY33* (Sun et al., 2019). VvERF113 enhances the waterlogging tolerance of *Arabidopsis* by interacting with HD-Zip I transcription factor VvATHB-13 (Min et al., 2026). *VvERF111* regulates chlorophyll degradation by activating the expression of *VvCLH1*, thereby contributing to rachis browning in grapes (Zou et al., 2023).

Herein, a *VvERF105* gene was isolated from the drought-tolerant grape cv. ‘Thompson Seedless’, which is widely cultivated under the arid, continental climate of China’s Turpan Basin, receiving an annual average rainfall of less than 20 mm. Unlike *FaERF105*, which was mainly expressed in roots and stolons of strawberry (Li et al., 2026), the expression level of the *VvERF105* gene was highest in mature leaves and lowest in berries, indicating that its expression exhibited tissue space-time specificity. This divergence suggests possible neofunctionalization after species separation, where grapevine prioritizes stress defense in photosynthetic leaves rather than early fruit development. VvERF105 has the same nuclear localization as the homologues of strawberry (Li et al., 2026) and *Arabidopsis* (Bolt et al., 2017; Illgen et al., 2020), and they respond quickly to drought and low temperature treatments, indicating that *VvERF105* could be used as an early response factor in the signal transduction and stress response process of drought and low temperature stresses. In contrast, *VvERF105* was not involved in the positive regulation of high temperature stress. Moreover, it could be strongly induced under exogenous ABA treatment. These findings suggested that *VvERF105* is a stress-related transcription factor preferentially expressed in mature leaves, responds to drought and low temperature rather than high temperature, and potentially acts downstream of ABA signaling pathway.

Phenotypic observations showed that *VvERF105* positively regulates grape drought resistance. *FaERF105* has a similar function in strawberry resistance to drought stress (Li et al., 2026). Notably, the stomatal aperture of *VvERF105* mutant plants after drought treatment in this study was significantly smaller than that of WT plants. This finding was unlike that of most drought-resistant genes that improve drought resistance by regulating stomatal closure (Cui et al., 2022; Zhao et al., 2025). Stomatal regulation is the central mechanism for balancing water conservation and photosynthetic efficiency (Mohammad et al., 2025). Nevertheless, we hypothesize that the narrowed stomatal pores of mutants may overly restrict CO_2_ influx, thereby suppressing the production of photosynthates (Chen et al., 2022). Deficient energy supply consequently impairs the synthesis of osmoprotectants (e.g., proline) and the activation of antioxidant systems, rendering mutants more susceptible to drought stress. Proline content, MDA content, POD, SOD, and CAT activities could be used as biochemical indicators for the identification of drought tolerance in multiple plants (Liu et al., 2023). Results showed that the loss of *VvERF105* function leads to a decrease in osmotic regulation ability, an increase in membrane lipid peroxidation, and a decrease in active oxygen scavenging ability under drought stress, which leads to more severe cell damage and physiological metabolic disorders. Meanwhile, the deletion of *VvERF105* will lead to the inhibition of the expression of some drought response genes like *VvDREB2A*, *VvERD14*, *VvKIN2*, *VvNCED1*, *VvRD22*, and *VvRD29B*. This phenomenon indicated that *VvERF105* positively regulates the transcriptional expression of multiple downstream drought response genes and participates in drought resistance regulation by activating the antioxidant system, thereby promoting the synthesis of osmotic adjustment substances, which maintain the cell membrane stability. The positive regulatory function of *VvERF105* in response to drought resistance in grape was fully verified at the molecular and physiological levels.

Yeast two-hybrid, BiFC, and Co-IP assays confirmed that VvERF105 interacted with the SnRK family protein kinase VvSnRK1. As a central regulator of energy perception and metabolic integration in plants (Kuang et al., 2026), *SnRK1* plays a central regulatory role in plant ABA signaling, energy metabolism, and drought stress response (Zeng et al., 2026). It participates in stress response by phosphorylating downstream transcription factors (Jossier et al., 2009; Chen et al., 2021). Previous studies have shown that in *Arabidopsis*, SnRK1 kinase directly or indirectly binds to and phosphorylates a variety of target proteins, including metabolic enzymes, regulatory proteins and transcription factors (Baena-González et al., 2007; Gutierrez-Beltran and Crespo, 2022). High levels of sucrose can induce stomatal closure (Kottapalli et al., 2018). The interaction between ABA and sugar signaling regulates the production of systemic leaf stomata by controlling sucrose transport (Yao et al., 2025). In citrus fruit, ABA signaling orchestrates SnRK1 phosphorylates and regulates transcription factors WRKY41 to regulate sugar accumulation under drought stress (Zeng et al., 2026). Based on these findings, we speculate that SnRK1 acts as a key integrator of sugar and ABA signals to modulate stomatal aperture under drought conditions. However, the interaction between VvSnRK1 and VvERF105 appears to operate through a distinct mechanism, as elaborated below. *In vitro* phosphorylation experiments revealed that VvSnRK1 did not directly phosphorylate VvERF105, suggesting that they were not typical kinase-substrate phosphorylation regulation modes despite forming protein complexes *in vivo*. Therefore, we propose that VvSnRK1 regulates VvERF105 through a phosphorylation-independent mechanism. Non-catalytic regulation of transcription factors by kinase has been documented. For instance, the interaction between ZmCCaMK and ZmWRKY104 improved salt tolerance in maize, but ZmCCaMK did not phosphorylate ZmWRKY104. Instead, they synergistically activated antioxidant defense via a novel mechanism to enhance brassinosteroid-induced salt tolerance in maize (Zhao, 2026). Similarly, VvSnRK1 may influence VvERF105 subcellular localization, protein stability, or recruitment of co-regulators. These possibilities remain to be experimentally verified, yet we establish a rational framework for deciphering the functional significance of the VvERF105-VvSnRK1 complex. Importantly, this non-catalytic interaction establishes a direct molecular link between cellular energy status and VvERF105 activity, operating in an ABA-independent manner within the VvERF105-specific context, although SnRK1 itself is known to engage in both ABA-dependent and -independent pathways. Under drought stress, declining photosynthesis and sugar depletion activate SnRK1. Activated SnRK1 can interact with VvERF105, potentially fine-tuning its transcriptional output in accordance with cellular energy balance. Such a mechanism could prevent the plant from mounting a full stress response when energy reserves are critically low, avoiding metabolic collapse.

### 4.1 Conclusion

**Figure 7:**
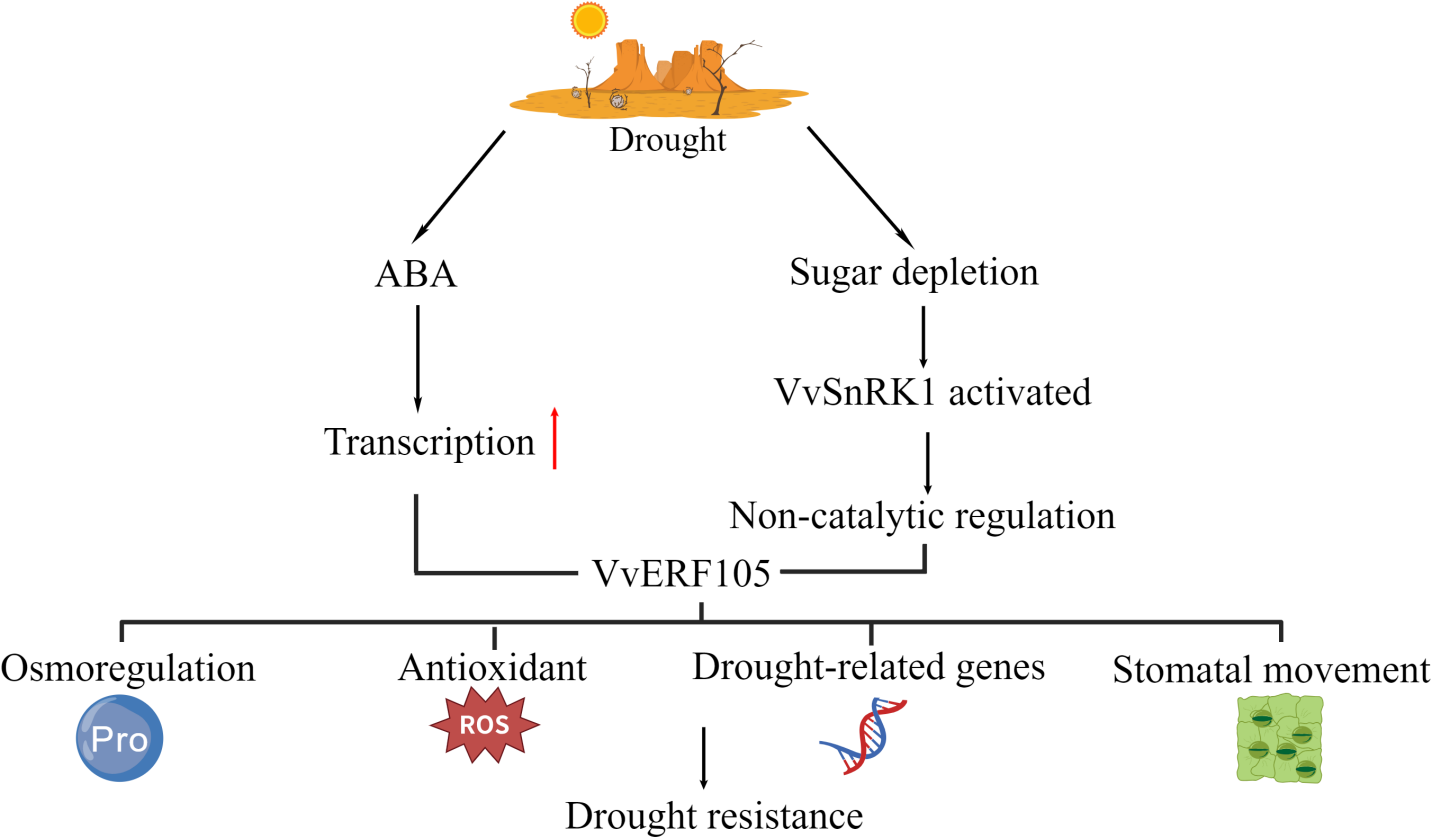
A hypothetical model of *VvERF105*-mediated drought response in grape. Under drought stress, ABA induced the expression of *VvERF105*. At the same time, energy stress activates VvSnRK1, which interacts with VvERF105 protein in a non-catalytic manner (independent of phosphorylation), and fine-tunes the activity of VvERF105 according to the cell energy state. The two pathways converge on *VvERF105* to jointly regulate stomatal aperture, downstream osmoregulation substance content, antioxidant enzyme activity and drought resistance-related genes, and ultimately enhance the drought resistance of grapes.

*VvERF105* is a positive regulator of drought resistance in grape, preferentially expressed in mature leaves and responsive to drought stress as well as low and high temperature. Our results demonstrate that *VvERF105* acts downstream of ABA signaling, as its transcript levels are strongly induced by exogenous ABA. Furthermore, VvERF105 physically interacts with the energy sensor VvSnRK1, and this interaction does not involve direct phosphorylation of VvERF105 by VvSnRK1. Thus, VvERF105 is positioned downstream of SnRK1-mediated energy signaling through a non-catalytic, phosphorylation-independent mechanism. We propose that *VvERF105* serves as a molecular hub integrating ABA-dependent and ABA-independent drought responses: ABA controls its transcriptional abundance, while SnRK1 finetunes its activity according to cellular energy status. Functionally, *VvERF105* may contribute to drought tolerance through effects on osmotic regulation, the antioxidant system, and the expression of downstream drought-related genes. Editing of *VvERF105* led to decreased stomatal aperture under drought stress, indicating that *VvERF105* is involved in regulating stomatal movement. Overall, *VvERF105* represents a candidate gene for breeding drought-resistant grape varieties, and the proposed integration of ABA and energy signaling provides a mechanistic framework for future investigation (**Figure 7**).

## Data Availability

The original contributions presented in the study are included in the article/**Supplementary Material**. Further inquiries can be directed to the corresponding authors.

## Supplementary material

Supplementary Figure 1 Acquisition of *VvERF105* gene-edited grapes. **(A)** Schematic diagram of the sgRNA expression cassettes and pYLCRISPR/Cas9Pubi-N vector of *VvERF105* gene. T1 denotes target 1, and T2 denotes target 2. **(B)** PCR reaction results for constructing a single target vector of the *VvERF105* gene. Lane 1: DNA marker; lane 2: the first PCR reaction for target 1; lane 3: the first PCR reaction for target 2; lane 4: the second PCR reaction for target 1; lane 5: the second PCR reaction for target 2. **(C)** sgRNA expression cassette of a single-target vector. Lane 1: DNA marker; lane 2: detection result for target 1; lane 3: detection result for target 2. **(D)** The connection between the sgRNA expression cassette and pYLCRISPR/Cas9Pubi-N vector. Lane 1 is DNA marker, lanes 2, 4, 6, 8, 10, and 12 used SP-R and AtU3dT1 as detection primers, while lanes 3, 5, 7, 9, 11, and 13 used gRT1 and SP-L1 as detection primers. **(E)** Transgenic flow chart. **(F)** Sequence analysis of the CRISPR/Cas9 target sites in the *VvERF105*-edited grape lines. 1T1: the first target of line T1, 1T2: the first target of line T2, 1T3: the first target of line T3, 2T1: the second target of line T1, 2T2: the second target of line T2, 2T3: the second target of line T3.

Supplementary Figure 2 Detection of transcriptional self-activation of VvERF105 in yeast. The bait construct pGBKT7-*VvERF105* was transformed into the yeast strain and plated on SD/-Trp/-Leu medium containing 200 μg/mL AbA and X-α-gal. Neither growth nor blue color was observed for pGBKT7-*VvERF105*, indicating that VvERF105 lacks self-activation activity. The positive control (pGBKT7-53 + pGADT7-T) grew well and turned blue, while the negative control (pGBKT7-Lam + pGADT7-T) did not grow, confirming the functionality of the system.

Supplementary Figure 3 Phosphorylation assay *in vitro*. The phosphorylation products separated by Phos-tag SDS-PAGE **(A)** and conventional SDS-PAGE **(B)** were detected via Coomassie Brilliant Blue staining. The reaction systems contained *VvERF105*-HIS and *VvSnRK1*-GST with or without ATP supplementation. In the Phos-tag gel **(A)**, the migration rate of the *VvERF105*-HIS band showed no difference in the presence and absence of ATP, indicating that VvSnRK1 did not induce detectable phosphorylation of VvERF105. The conventional gel **(B)** confirmed equal protein loading in all lanes.
