# Supplementary figures and images for: VvERF105 enhances drought resistance in grape through interaction with VvSnRK1
Source: Front Plant Sci. 2026 Jul 9;17:1884274. doi: 10.3389/fpls.2026.1884274 (PMC13391271; doi:10.3389/fpls.2026.1884274)

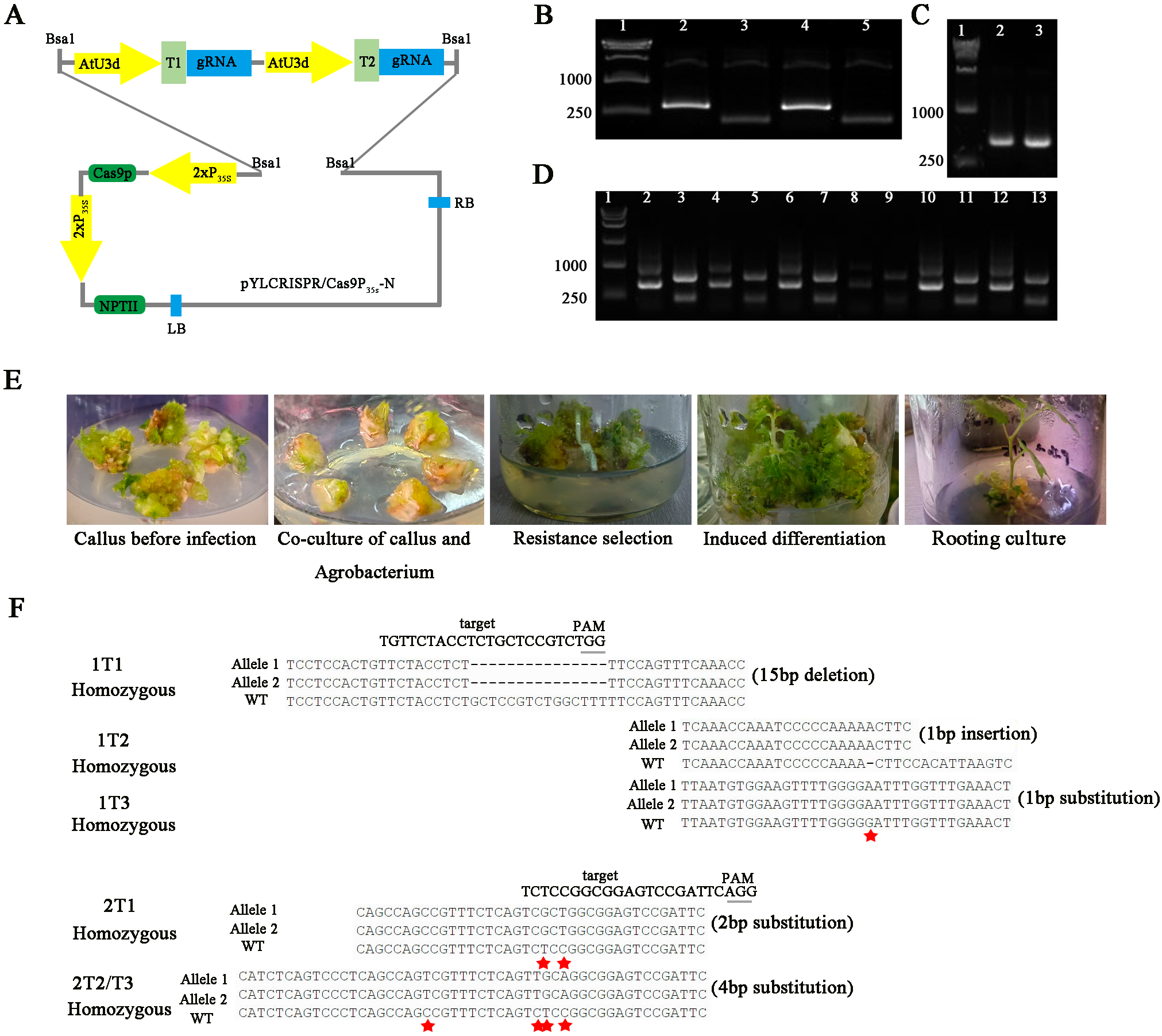

Supplement: Supplementary Figure 1 — Acquisition of VvERF105 gene-edited grapes. (A) Schematic diagram of the sgRNA expression cassettes and pYLCRISPR/Cas9Pubi-N vector of VvERF105 gene. T1 denotes target 1, and T2 denotes target 2. (B) PCR reaction results for constructing a single target vector of the VvERF105 gene. Lane 1: DNA marker; lane 2: the first PCR reaction for target 1; lane 3: the first PCR reaction for target 2; lane 4: the second PCR reaction for target 1; lane 5: the second PCR reaction for target 2. (C) sgRNA expression cassette of a single-target vector. Lane 1: DNA marker; lane 2: detection result for target 1; lane 3: detection result for target 2. (D) The connection between the sgRNA expression cassette and pYLCRISPR/Cas9Pubi-N vector. Lane 1 is DNA marker, lanes 2, 4, 6, 8, 10, and 12 used SP-R and AtU3dT1 as detection primers, while lanes 3, 5, 7, 9, 11, and 13 used gRT1 and SP-L1 as detection primers. (E) Transgenic flow chart. (F) Sequence analysis of the CRISPR/Cas9 target sites in the VvERF105-edited grape lines. 1T1: the first target of line T1, 1T2: the first target of line T2, 1T3: the first target of line T3, 2T1: the second target of line T1, 2T2: the second target of line T2, 2T3: the second target of line T3. [file Image1.tif]

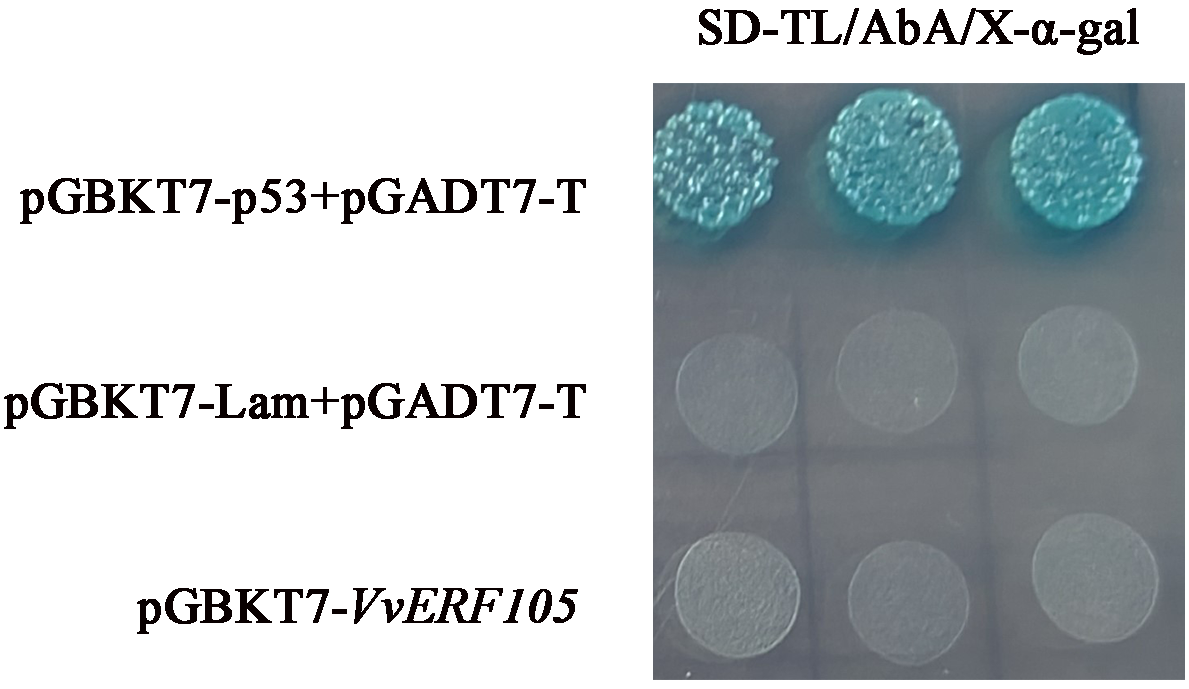

Supplement: Supplementary Figure 2 — Detection of transcriptional self-activation of VvERF105 in yeast. The bait construct pGBKT7-VvERF105 was transformed into the yeast strain and plated on SD/-Trp/-Leu medium containing 200 μg/mL AbA and X-α-gal. Neither growth nor blue color was observed for pGBKT7-VvERF105, indicating that VvERF105 lacks self-activation activity. The positive control (pGBKT7-53 + pGADT7-T) grew well and turned blue, while the negative control (pGBKT7-Lam + pGADT7-T) did not grow, confirming the functionality of the system. [file Image2.tif]

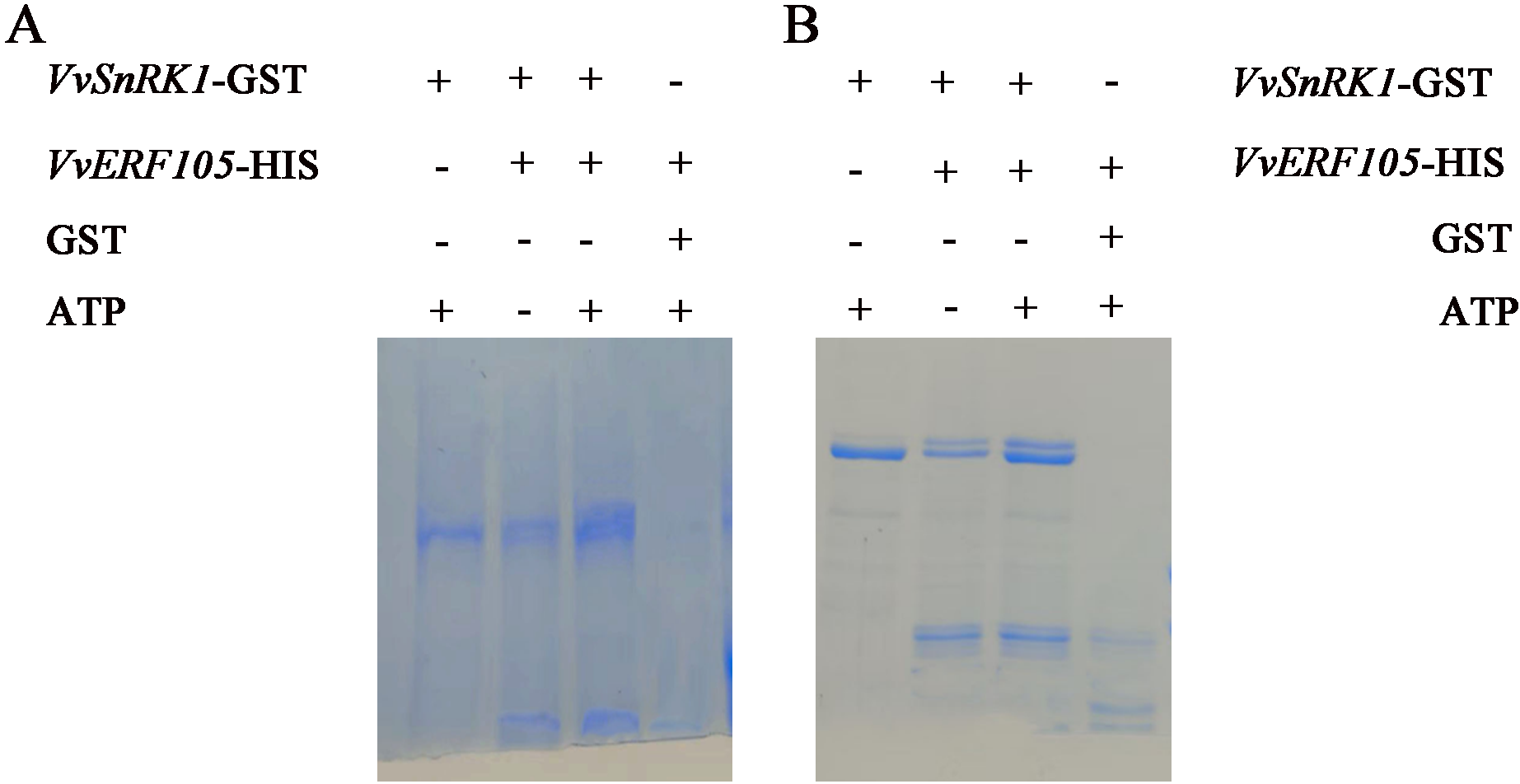

Supplement: Supplementary Figure 3 — Phosphorylation assay in vitro. The phosphorylation products separated by Phos-tag SDS-PAGE (A) and conventional SDS-PAGE (B) were detected via Coomassie Brilliant Blue staining. The reaction systems contained VvERF105-HIS and VvSnRK1-GST with or without ATP supplementation. In the Phos-tag gel (A), the migration rate of the VvERF105-HIS band showed no difference in the presence and absence of ATP, indicating that VvSnRK1 did not induce detectable phosphorylation of VvERF105. The conventional gel (B) confirmed equal protein loading in all lanes. [file Image3.tif]
