# Supplementary material for: VvERF105 enhances drought resistance in grape through interaction with VvSnRK1
Source: Front Plant Sci. 2026 Jul 9;17:1884274. doi: 10.3389/fpls.2026.1884274 (PMC13391271; doi:10.3389/fpls.2026.1884274)
Supplement: Supplementary file 4 [file Table1.doc]

**Table S1 Primer sequences used in the study (5'-3')**

| Primer name | sequence |
| --- | --- |
| *VvERF105*-F | ATGGCAGAAGAAGTTTCATCGGTTCACTTCA |
| *VvERF105*-R | TCAAACTATAAGCTGAGAATATGCTATCCAAGGAT |
| qRT-*VvERF105*-F | GGCCAGTCATGGGGTTAGTCCGT |
| qRT-*VvERF105*-R | AACGGCGTTAACGGTGGCAGA |
| qRT-*VvActin7*-F | TTCTCGTTGAGGGCTATTCCA |
| qRT-*VvActin7*-R | CCACAGACTTCATCGGTGACA |
| *VvERF105*-gRT1 | TGTTCTACCTCTGCTCCGTCgttttagagctagaaat |
| *VvERF105*-AtU3dT1 | GACGGAGCAGAGGTAGAACATgaccaatggtgctttg |
| *VvERF105*-gRT2 | TCACCGGCGGAGTCCGATTCgttttagagctagaaat |
| *VvERF105*-AtU3dT2 | GAATCGGACTCCGCCGGTGATgaccaatggtgctttg |
| U-F | CTCCGTTTTACCTGTGGAATCG |
| gR-R | CGGAGGAAAATTCCATCCAC |
| Pps-R | TTCAGAGGTCTCTACCGACTAGTCACGCGTATGGAATCGGCAGCAAA |
| Pgs-2 | AGCGTGGGTCTCGTCAGGGTCCATCCACTCCAAGCTC |
| Pps-2 | TTCAGAGGTCTCTCTGACACTGGAATCGGCAGCAAAGG |
| Pgs-L | AGCGTGGGTCTCGCTCGACGCGTATCCATCCACTCCAAGC |
| SP-L1 | GCGGTGTCATCTATGTTACTAG |
| SP-R | TGCAATAACTTCGTATAGGCT |
| *VvERF105*-target-F | GTCTCTCATGTAAATGATCCATCCCA |
| *VvERF105*-target-R | GCTTTGGAGCCACGCATCTCA |
| qRT-*VvRD22*-F | TGGCCCTTGGTATTGCCAAACACT |
| qRT-*VvRD22*-R | CGCCTATATCAGTCCCTGGGCG |
| qRT-*VvKIN2*-F | ACTGAGAAGATGATGGACAAGG |
| qRT-*VvKIN2*-R | TTGTTCATTCCAGTTGCATCCT |
| qRT-*VvDREB2A*-F | CGGGAGCCAAACAGAGGGAGT |
| qRT-*VvDREB2A*-R | CATGGCCCTTGCAGCTTCGTC |
| qRT-*VvNCED1*-F | CAGCCGTGGCTCTCCTGTGG |
| qRT-*VvNCED1*-R | TCGGTCTCCGGCTCTTCCCA |
| qRT-*VvRD29B*-F | GGACACTTCAAGGCAGTATGG |
| qRT-*VvRD29B*-R | TCTCGTTGGATGGCTTCTCA |
| qRT-*VvERD14*-F | TGATGAAGTAGTTCCACCACAG |
| qRT-*VvERD14*-R | TTCTTCTCTTCTTCGGTCTTGG |
| *VvERF105*-GFP-F | ACGGGGGACGAGCTCGGTACCATGGCAGAAGAAGTTTCATCGGTTCACTTCA |
| *VvERF105*-GFP-R | GGTGTCGACTCTAGAGGATCCTCAAACTATAAGCTGAGAATATGCTATCCAAGGAT |
| 35S | ATCCTTCGCAAGACCCTTCCTCTAT |
| SeqYFPR | CAGGGTCAGCTTGCCGTAG |
| T7 | GTAATACGACTCACTATAGGGCGA |
| 3'BD | TTTTCGTTTTAAAACCTAAGAGTC |
| 3'AD | AGATGGTGCACGATGCACAG |
| pGBKT7-*VvERF105*-F | ATGGCCATGGAGGCCGAATTCATGGCAGAAGAAGTTTCATCGGTTCACTTCA |
| pGBKT7-*VvERF105*-R | CGCTGCAGGTCGACGGATCCTCAAACTATAAGCTGAGAATATGCTATCCAAGGAT |
| pGADT7-*VvSnRK1*-F | GCCATGGAGGCCAGTGAATTCATGGGAAATGCTAATGGCCGAGAG |
| pGADT7-*VvSnRK1*-R | CCGTATCGATGCCCACCCGGGCCTTTTCAGTGGCTTATAAAGGACAACAG |
| pEYFP-C-*VvSnRK1*-F | gggactctagaggatctcgagATGGGAAATGCTAATGGCCGAGAG |
| pEYFP-C-*VvSnRK1*-R | ATCGTATGGGTACATGGTACCCCTTTTCAGTGGCTTATAAAGGACAACAG |
| pEYFP-N-*VvERF105*-F | AGTGGATCCGTCGACCTCGAGATGGCAGAAGAAGTTTCATCGGTTCACTTCA |
| pEYFP-N-*VvERF105*-R | CTCCTACCCGGGAGCGGTACCTCAAACTATAAGCTGAGAATATGCTATCCAAGGAT |
| pBI221-F | CTATCCTTCGCAAGACCCTTCCT |
| pBI221-R | GATAATCATCGCAAGACCGGCAAC |
| *VvERF105*-HA-F | GGGGACAACTTTGTACAAAAAAGTTGGCATGGCAGAAGAAGTTTCATCG |
| *VvERF105*-HA-R | GGGGACAACTTTGTACAAGAAAGTTGGGCAAACTATAAGCTGAGAATATGCTATCCAAGG |
| *VvSnRK1*-Flag-F | GGGGACAACTTTGTACAAAAAAGTTGGCATGGGAAATGCTAATGGCC |
| *VvSnRK1*-Flag-R | GGGGACAACTTTGTACAAGAAAGTTGGGCACCTTTTCAGTGGCTTATAAAGGAC |
| *VvSnRK1*-GST-F | gatctggttccgcgtggatccATGGGAAATGCTAATGGC |
| *VvSnRK1*-GST-R | tcagtcagtcacgatgcggccgcTTACCTTTTCAGTGGCTT |
| *VvERF105*-HIS-F | acagcaaatgggtcgcggatccATGGCAGAAGAAGTTTCA |
| *VvERF105*-HIS-R | gtggtggtgctcgagtgcggccgcTCAAACTATAAGCTGAGA |
