# Supplementary material for: VvERF105 enhances drought resistance in grape through interaction with VvSnRK1
Source: Front Plant Sci. 2026 Jul 9;17:1884274. doi: 10.3389/fpls.2026.1884274 (PMC13391271; doi:10.3389/fpls.2026.1884274)
Supplement: Supplementary file 5 [file Table2.doc]

**Table S2 Accession numbers of proteins used for phylogenetic analysis**

| Protein name | Accession number |
| --- | --- |
| TSERF105 | UZY20452.1 |
| VaERF080 | AYV61529.1 |
| PNERF105 | XP_002281912.1 |
| VrERF5-like | XP_034711512.1 |
| ZmERF5 | PWZ39589.1 |
| PmERF105 | XP_008234445.1 |
| PpERF105 | XP_007218706.1 |
| MdERF105 | NP_001315890.1 |
| PcERF105-like | NC_084807.1 |
| OsERF105 | XP_015635116.1 |
| TaERF105-like | XP_044328607.1 |
| CsERF6 | ADW09319.1 |
| AtERF105 | CAO7056047.1 |
| MaERF105-like | XP_009389354.2 |
| NnERF105-like | XP_010275423.1 |
| BpERF13 | QIJ58756.1 |
| TcERF105 | XP_017973866.1 |
| HsERF105-like | KAE8681444.1 |
| AcERF5-like | PSS33215.1 |
